# Collection of Ambulatory Electrocardiogram and Behavioral Data for the Identification of Digital Biomarkers for Heart Failure (CATCH-ECG): Protocol for a Prospective Cohort Study

**DOI:** 10.2196/79651

**Published:** 2025-12-19

**Authors:** Jakob Eyvind Bardram, Gouthamaan Manimaran, Chloë Cammaerts, Sadasivan Puthusserypady, Helena Dominguez

**Affiliations:** 1 Department of Health Technology Technical University of Denmark Kgs. Lyngby Denmark; 2 Technical University Hospital of Copenhagen Copenhagen Denmark; 3 Department of Cardiology Bispebjerg and Frederiksberg Hospitals Copenhagen Denmark; 4 Copenhagen University Hospital Copenhagen Denmark

**Keywords:** heart failure, digital phenotyping, ambulatory electrocardiogram monitoring, electrocardiogram, ECG, mobile health, mHealth, wearables, cohort study

## Abstract

**Background:**

Heart failure (HF) is a complex clinical syndrome with a high morbidity and mortality rate. Despite advancements in treatment, the recurrence of HF remains a significant challenge, often leading to deteriorating health conditions and increased pressure on the health care system. Early detection of recurrence is pivotal in mitigating and managing the adverse outcomes associated with HF.

**Objective:**

The primary objective of this study is to collect data to facilitate the identification of digital biomarkers that may indicate deterioration of the heart and, ultimately, develop algorithms that can predict HF.

**Methods:**

This prospective cohort study is conducted in Copenhagen, Denmark, and will recruit individuals diagnosed with decompensated HF. Participants will be followed for a period of 1 year, during which they will undergo a quarterly assessment period every 3 months. Each quarterly assessment period spans 7 days and involves continuous monitoring using an ambulatory electrocardiogram sensor. Throughout each quarterly assessment period, participants will also complete daily assessments and questionnaires. All data will be collected using a dedicated mobile app installed on the participants’ personal smartphones and securely stored in a cloud-based system.

**Results:**

This study is part of the Cardio-Share Model for Cross-Sectoral Ambulatory Treatment of Congestive Heart Disease Based on Personal Health Technology project. Technical and regulatory preparation started in 2023. Recruitment for this study started in January 2025 and is expected to be completed by the end of 2026. The dataset will be anonymized and published for further research.

**Conclusions:**

This study aims to provide a comprehensive longitudinal open-source dataset of HF recorded in real-world ambulatory conditions that enhances our understanding of HF signs and symptoms. This dataset will provide an important source for detailed analysis and understanding of HF based on ambulatory and contextual physiological data. Such insight has the potential to enhance the clinical management of individuals with HF and enable them to handle their condition at home.

**International Registered Report Identifier (IRRID):**

DERR1-10.2196/79651

## Introduction

Heart failure (HF) remains an increasing global health threat despite improvements in diagnostic and therapeutic methods. Due to an aging population and improved care and life expectancy of patients, the burden of HF on the health care system is increasing in Denmark and elsewhere, wherein HF alone accounted for 2% of Danish total health expenditure. Approximately 8% of patients with HF succumb within 1 year of diagnosis in Denmark [1], whereas 50% of patients diagnosed with HF still pass away within 5 years [2,3]. By 2030, it is estimated that, within the age group of 65 to 70 years, the incidence of HF will increase to 8.5% from 4.3% in 2012 [4,5] (ie, an unprecedented 50% increase in a little less than 2 decades). The high morbidity and mortality associated with HF may, in part, stem from discrepancies between the treatment prescribed by the physician and the regimen actually followed by the patient [6]. Additionally, delays in reporting noticeable HF symptoms to a general practitioner may result in missed opportunities for timely and effective intervention [7].

It has been suggested to address the growing prevalence of HF incidence and the lack of efficient treatment using mobile health (mHealth) technology [8]. Access to mHealth technologies such as ambulatory electrocardiogram (ECG) sensors and HF-specific smartphone apps seems to reduce mortality and HF-related hospitalizations and, thus, may reduce the burden of HF on the health care system. Studies have shown that the use of telemedicine and remote monitoring of patients with HF is associated with a reduced risk of cardiovascular-related mortality [9,10] and a reduction in rehospitalization [11-14]. Thus, according to the European Society of Cardiology guidelines, telemonitoring of patients with HF may be considered a class IIb indication [15].

However, most studies investigate the outcome of the intervention and do not investigate whether the collected data can be used for real-time monitoring of disease worsening or prediction of HF. In contrast to CHA_2_DS_2_-VASc [16] and other scores for assessing risk of stroke, there are currently no scores that allow for prediction of worsening of HF. Moreover, telemonitoring typically only collects very coarse-grained data such as symptoms, weight, heart rate (HR), and blood pressure, and whether wearable technologies for detailed monitoring of heart rhythm offer additional benefits is uncertain [15].

The purpose of this study is to collect a “rich” ambulatory dataset on HF. The aim is to collect data over a long period (1 year) in the free-living and daily environment of the patient in a very detailed manner. This would enable the detection of HF worsening before clinical symptoms are present.

The collected data include both physiological (ECG) and movement sensor data from a heart monitor, contextual and behavioral data from the sensor in the smartphone, and a series of patient-reported data based on experience sampling and surveys on a daily and weekly basis.

The data collected in this study will then be used for the identification of digital biomarkers, which can be used for continuous monitoring of the cardiovascular condition of the patient in real time and provide the input for algorithmic models for predicting disease deterioration. This study is part of the Cardio-Share Model for Cross-Sectoral Ambulatory Treatment of Congestive Heart Disease Based on Personal Health Technology (CATCH) project and, thus, is named the CATCH-ECG study.

## Methods

### Study Design and Participants

This study is a prospective observational cohort investigation involving the enrollment of 40 individuals with a clinical diagnosis of HF residing either in their own homes or in nursing facilities. Participants are recruited from the outpatient population receiving treatment for HF decompensation at the Department of Cardiology, Bispebjerg-Frederiksberg Hospital (BFH), within the Capital Region of Denmark (RegionH). Eligibility for inclusion is determined according to the criteria outlined in Textbox 1.

**Inclusion and exclusion criteria.**

**Inclusion criteria**

- Participant age of >50 years
- Diagnosis of heart failure (HF) with any of the following International Classification of Diseases (ICD) codes: I50.40—unspecified combined systolic and diastolic (congestive) HF, I50.20—unspecified systolic (congestive) HF, I50.30—unspecified diastolic (congestive) HF, I50.42—chronic combined systolic and diastolic (congestive) HF, and I50.9—HF (unspecified)
- Presence of at least one of the following: hypertension, diabetes, coronary disease, prior clinical ischemic cerebral attack (stroke or transient ischemic attack), or silent infarcts observed in brain imaging
- Willingness to use a smartphone app and chest-worn electrocardiogram (ECG) monitor for 1 year
- Participants displaying symptoms suggestive of HF, such as dyspnea, orthopnea, paroxysmal nocturnal dyspnea, or edema

**Exclusion criteria**

- Participants receiving palliative care with severe or end-stage HF (ICD code I50.84)
- Participants with a pacemaker or implantable cardioverter-defibrillator
- Life expectancy under 1 year
- Other significant cardiac conditions that could confound ECG recordings, such as arrhythmias requiring intervention
- Participants who may not be able to consistently provide data or adhere to study protocols due to cognitive, logistical, or other reasons and unable to read and understand Danish or English

Participants are enrolled on a rolling basis. The target age range is 50 to 80 years, with a normal distribution and an equal number of men and women, and recruitment is limited to a European population due to the low sample size requirement. Recruitment started in the first quarter of 2025, with plans to complete data collection by the end of 2026.

### Sample Size

The primary objective of this study is data collection for algorithm development rather than hypothesis testing. Therefore, the sample size is not obtained from a power calculation but rather to ensure a diverse range of data points; capture variations; and ensure sufficient representation for subgroups based on age, gender, and other demographic factors. The target sample size is 40 participants. Hence, we aim to contact approximately 70 participants given an approximate conversion rate of 60%.

### End Points

The following end points are needed to develop algorithms that can predict worsening HF. The primary end point is information on death and rehospitalizations.

The secondary end point is worsening of HF, determined based on patient readmission with decompensated HF and quality of life (QoL), which are assessed using the following repeated questionnaires throughout the study: Fatigue Assessment Scale (FAS) [17] and EQ-5D-5L [18].

### Study Timeline

**Figure 1:**
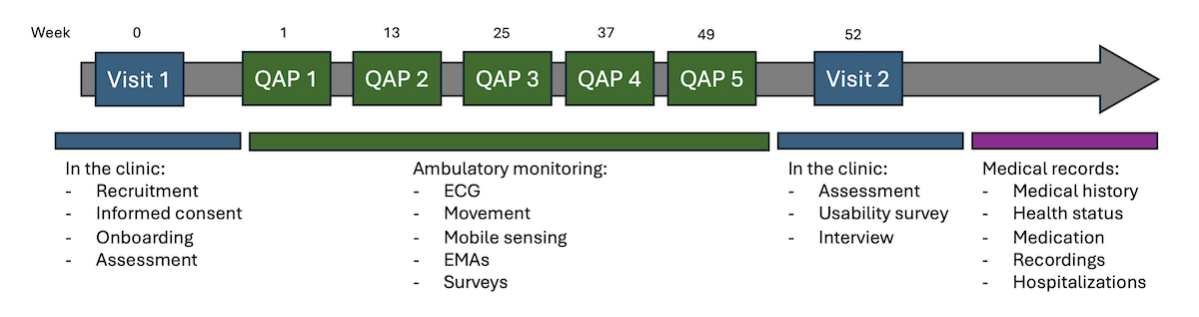
The main timeline of the study, showing the clinical phases (blue), the ambulatory monitoring phase (green), and the clinical data collection phase (purple). The study runs for 1 year (52 weeks) for each participant and includes 2 clinical visits (onboarding and offboarding) and 5 quarterly assessment periods (QAPs), each lasting 1 week. ECG: electrocardiogram; EMA: ecological momentary assessment.

The timeline of the study is shown in Figure 1. The study visits are conducted at baseline (week 1) and at the end of the study (week 52) in the clinic at BFH.

During visit 1 (onboarding), participants receive detailed information about the study and provide written informed consent. The study app is then installed on the participants’ personal smartphones, and they are issued an ambulatory ECG sensor. Participants are guided through the setup process, including logging into the app, enrolling in the study, accessing study-related information, completing assigned tasks, pairing the ECG device with their smartphone, and replacing the sensor battery if necessary.

As part of the onboarding visit, participants use the study app to fill in an onboarding survey collecting information about demographics, current cardiovascular diseases or problems, and smoking and drinking habits, as well as the FAS [17] and the EQ-5D-5L (QoL) [18] questionnaires. The participants are also helped to mount the device on their chest, connect it to the smartphone, and see the data being recorded. All of these activities also work as training in using the app and the sensor.

During the ambulatory monitoring phase, participants engage in 5 quarterly assessment periods, each lasting up to 1 week (7 days). Each quarterly assessment period is initiated through a phone call from the researchers.

During each quarterly assessment period, data are collected from the ECG sensor and the smartphone, as outlined in Table 1. This includes passive sensing data from the smartphone (top part of Table 1); electrophysiological and movement data from the ECG sensor; ecological momentary assessments (EMAs) on symptoms, sleep, stress, weight, and general health; and the FAS and EQ-5D-5L questionnaires. At the end of each quarterly assessment period, participants no longer need to wear the ECG sensor and can store it safely for the next quarterly assessment period. They can optionally continue to record their symptoms on the app. There are no clinical in-person visits during the ambulatory monitoring phase; all communication between the researchers and participants is done via the app or phone.

**Table 1:** Data features collected on the CARP (Copenhagen Research Platform) Studies app with source and sampling rate.

Source and parameter		Type of data collection	Sampling rate
**Phone**			
	Device information	Sensed	Once
	Battery	Sensed	EB^a^
	Screen	Sensed	EB
	Location	Sensed	EB
	Steps	Sensed	EB
	Activity	Sensed	EB
	Weather	Sensed	30 min
	Air quality	Sensed	30 min
**ECG^b^ sensor**			
	Device information	Sensed	On connection
	HR^c^	Sensed	EB
	ECG	Sensed	EB
	IMU^d^	Sensed	EB
	Device tap	PR^e^	EB
**Participant**			
	Symptoms	PR	EB
	Sleep	PR	Daily
	Stress	PR	Daily
	Weight	PR	Weekly
	General health	PR	Weekly
	Onboarding	PR	Once
	Fatigue Assessment Scale	PR	Weekly
	EQ-5D-5L	PR	Weekly

^a^EB: event-based.

^b^ECG: electrocardiogram.

^c^HR: heart rate.

^d^IMU: inertial movement unit.

^e^PR: participant-reported.

On completion of the study, participants attend a second clinical visit (offboarding), during which they fill in the FAS and EQ-5D-5L questionnaires one last time. They also complete the Copenhagen Center for Health Technology Unified Methodology for Assessment of Clinical Feasibility (CUMACF) [19] questionnaire in an online survey platform (Google Forms) plus an interview. The semistructured interview is designed to complement the questions asked in the CUMACF questionnaire and covers motivation for participation, usability, user-friendliness, acceptance, trust perceptions, and suggestions for improvements. A researcher interviews the participant either in the clinic or over the phone. This interview lasts no more than 30 minutes and is audio recorded. The CUMACF questionnaire and the interview play no role in data collection strategies or algorithm deployments.

Immediately after the offboarding meeting, clinical data on the participant are extracted from the medical record at BFH. The medical data collected are listed in Table 2.

**Table 2:** Data extracted from the medical record.

Category	Variables
Demographic information	Age, gender, race and ethnicity, height, and weight
Medical history	Hypertension, diabetes, atrial fibrillation, chronic lung disease, and cardiovascular disease
Health status	Diagnosis, NYHA^a^ status, and HF^b^ observations
Medications	Current medications from the following list: ACEi^c^, ARB^d^, β-blockers, mineralocorticoids, neprilysin inhibitors, HCN^e^ channel blockers, nitrates, vasodilators, biguanides, loop diuretics, and thiazide diuretics
Cardiovascular measurements	Blood pressure, heart rate, ECG^f^, and LVEF^g^ measurements and recordings
Hospitalization and clinical visits	Hospital admissions and discharges, reason for hospitalizations, duration of stays, and cardiac procedures and surgeries

^a^NYHA: New York Heart Association.

^b^HF: heart failure.

^c^ACEi: angiotensin-converting enzyme inhibitor.

^d^ARB: angiotensin receptor blocker.

^e^HCN: hyperpolarization-activated cyclic nucleotide gated.

^f^ECG: electrocardiogram.

^g^LVEF: left ventricular ejection fraction.

### Ambulatory Data Collection System

**Figure 2:**
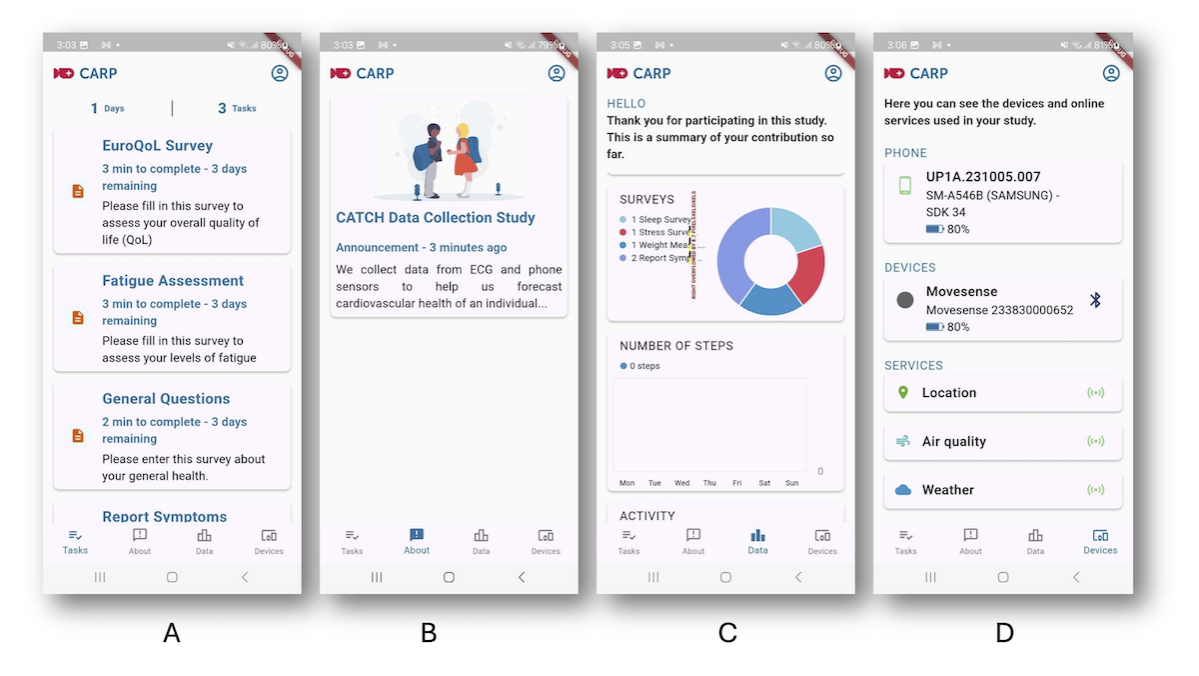
The main user interface of the CARP (Copenhagen Research Platform) Studies app: (A) the task list shows the tasks that the user needs to attend to, (B) the study page shows a description of the study plus a set of messages to the user from the researcher, (C) the data visualization page shows the data collected, and (D) the device page shows the devices (such as the Movesense electrocardiogram sensor) and online services being used.

Data are collected using the Copenhagen Research Platform (CARP) [20]. CARP is a full-stack platform for digital phenotyping consisting of a back-end cloud-based infrastructure for the management of studies, participants, and data combined with a smartphone-based study app called CARP Studies [21] designed for running digital phenotyping studies. The study app is available for both iPhones and Android phones from the Apple App Store and Google Play Store, respectively. When a study is initiated in CARP, it is defined by a study protocol, which details what data are to be collected. This study protocol is downloaded to the app when the participant signs in and accepts the study invitation. The main user interface of the CARP Studies app is shown in Figure 2, showing details from the CATCH-ECG study protocol.

The app contains a description of the study and what data are being collected (Figure 2B). The app prompts users to perform scheduled tasks (Figure 2A) during their participation in the study, as described in Table 1. Tasks include providing details on experienced symptoms when felt; answering daily EMAs on, for example, stress and sleep; and filling in weekly surveys on fatigue and QoL. Participants receive notifications from the app when a task needs attention. The app continuously collects sensor data in the background, such as battery consumption, geolocation information, step count, and physical activity (Table 1).

In addition to the app, a chest-worn ECG sensor is used to collect continuous ECG, HR, and inertial movement data in free-living conditions. The CARP Studies app has an extensible software architecture [22] that allows users to use different brands of ECG devices. This study uses Movesense HR2 sensors (Movesense Oy) [23] and Cortrium C3+ (Cortrium ApS) [24]. The ECG sensor is connected directly to the study app using Bluetooth (Figure 2D). Movesense HR2 is a sports sensor for measuring 1-lead ECG, HR, and movement using a 3D accelerometer and gyroscope on board. The sensor is light in comparison to alternatives (9.4 g) and has options for being mounted using a band around the torso or, alternatively, using an adhesive electrode. Cortrium C3+ is an approved medical sensor for measuring 3-lead ECG and movement via the onboard 3D accelerometer. The sensor is heavier and is mounted using 3 adhesive electrodes.

The data collected is visualized in the app as shown in Figure 2C. This includes all types of data, such as participant-reported data, sensor data collected from the phone, and physiological data collected from the ECG sensor.

### Data Collection

Table 1 summarizes the data types collected from the phone’s sensors, from the ECG sensor, and as participant-reported data (both EMAs and surveys). The data collected are intended to reveal information about the participant’s behavior (eg, screen activity and steps or inertial movement unit data), the environmental context (eg, location or weather), heart function (eg, ECG or symptoms), and general health and well-being (eg, sleep, stress, and fatigue assessments). It should be noted that this information originates from both sensor-based (“passive”) and participant-reported (“active”) data.

At the end of the study, as part of the offboarding visit, a usability assessment will be conducted using the CUMACF [19] method.

Table 2 summarizes the clinical data collected from the medical record. Clinical data are collected from a 5-year medical history when the study ends for each participant. These data provide a comprehensive view of the participants’ health status before and during the data collection phase.

### Data Management

Data during the study will be collected using the participants’ personal smartphones and uploaded to a secure cloud infrastructure located at the Technical University of Denmark. Data collection on the participants’ smartphones uses a randomly generated study-specific patient identifier. Clinical data on diagnosis, medical history, and medications, among other variables, will be collected at BFH and stored in a secure data storage platform (REDCap; Research Electronic Data Capture; Vanderbilt University [25]) in RegionH. Generation, transmission, storage, and analysis of health-related personal data during this study follow the European General Data Protection Regulation.

The Technical University of Denmark and RegionH are data controllers of their own data, and a shared data management agreement has been developed for sharing data in this study. The collected data will be kept as long as relevant for the research purpose of the study from which they were collected. This study does not share personal data with anyone outside the study, and personal data will not be published. It is the aim to publish the CATCH-ECG dataset in a public repository. All data will only be published anonymously and will have no personal identifiers.

### Quality Assurance

Data are reviewed after each quarterly assessment period to monitor data quality, ensure compliance with tasks and proper data capture, and identify any usability or bugs in the system’s setup. Noncompliant participants are contacted by the research team to explore reasons and propose solutions. Participants receive onboarding guidance for proper ECG sensor placement and are able to check that their HR is being detected correctly via the study app before starting the quarterly assessment period. While live signal quality monitoring is not performed during recording, all ECG data undergo detailed post hoc quality assessment using signal quality indexes and artifact detection during analysis.

A comprehensive report will be published summarizing the percentage of usable ECG and contextual data per participant and quarterly assessment period. Because ECG and smartphone sensors produce large, high-frequency datasets, moderate data loss is not expected to materially affect downstream analyses.

### Outcome Assessment

The primary objective of this study is to predict rehospitalization or death due to cardiovascular symptoms, as specified in the End Points section. Secondary target data include the outcomes (increase or decrease) from the FAS [17] and EQ-5D-5L [18] questionnaires.

### Data Analysis Plan

#### Digital Biomarker Discovery

This analysis focuses on leveraging ambulatory ECG data contextualized via actimetry sensor readings, smartphone-based activity detection, and participant-reported symptoms to identify anomalous cardiovascular patterns through statistical and machine learning methods. Known ECG-based risk indicators, particularly low HR variability [26,27], will be assessed across different contextual settings—rest, sleep, and intensive physical activities—to refine their prognostic capabilities regarding cardiac deterioration.

Further complex analyses will use machine learning techniques to detect both interpretable and black-box anomalies. For interpretable anomalies, multiple ECG-derived features such as P wave amplitude, QRS energy, and others will be computed. These features will be clustered together with contextual activity data to establish correlations with the primary and secondary study targets. Black-box anomaly detection will be conducted using advanced deep learning approaches, notably multi-instance learning, following methodologies similar to those proposed by Kamranfar et al [28].

#### Adaptive Risk Score Formulation

Another dimension of analysis will involve correlating established cardiovascular risk metrics—such as HR turbulence, HR recovery, and atrial fibrillation burden—with clinical end points and blood biomarkers, particularly kidney function and hemoglobin levels. The principal outcome of this analysis and of the broader dataset is the development of improved predictive risk scores for HF rehospitalization. These novel predictive scores will be benchmarked against traditional risk assessment tools, including the Framingham Risk Score and the pooled cohort equations, paving the way for a more personalized risk assessment.

Another important aspect of the collected data is the multiple recordings at quarterly assessment periods. Both the primary and secondary analyses will be conducted considering these repeated measures to observe the temporal evolution of digital biomarkers and risk scores as they approach the study end points. This approach aims to reveal whether a patient’s health is improving or declining, thus enabling medical practitioners to make timely decisions to prevent further health deterioration.

#### Model Development and Evaluation

Feature selection will be guided by both clinical interpretability and statistical relevance. Candidate features include ECG-derived measures (eg, HR variability, QRS duration and amplitude, and P wave morphology) and contextual smartphone or actimetry signals (eg, sleep, activity intensity, and HR recovery). Data-driven importance ranking will be performed using mutual information, recursive feature elimination, and Shapley Additive Explanations values derived from preliminary models. Model training will follow a nested cross-validation strategy with patient-level outer folds to avoid temporal or patient-level data leakage. Inner loops will be used for hyperparameter tuning and feature subset optimization.

Model performance will be reported using the area under the receiver operating characteristic curve, the area under the precision-recall curve, the *F*_1_-score, the Brier score, and calibration curves, with additional metrics (sensitivity and specificity) for binary classification tasks. Comparative analyses between interpretable and black-box models will be conducted to assess robustness and clinical utility.

#### Compliance, Usability, and Acceptance

Participants’ compliance is measured using logs from the study app, which reveal what tasks have been completed and which EMAs and surveys have been answered. Compliance with ECG monitoring during the quarterly assessment period is extracted from the collected data.

Usability and long-term acceptance of the app will be assessed based on the CUMACF surveys and by performing a thematic analysis of the interviews. Compliance thresholds are defined a priori: ≥72 hours of usable ECG signals per 7-day quarterly assessment period and a ≥60% EMA response rate are considered adherent. Nonetheless, any participant contributing data temporally linked to clinical end points will be retained in an *intention-to-include* analysis, recognizing that even partial data may reveal meaningful pre-event signal patterns.

### Ethical Considerations

The study will be conducted based on the principles expressed in the Declaration of Helsinki. The study, including the study protocol and all participant information materials, has been approved by the Scientific Ethics Committee for the Capital Region of Denmark (H-24052433).

Participants will receive both written and verbal information about the study. They will be given 24 hours to consider their participation and are encouraged to consult with a close relative such as a spouse or friend, if desired. Informed consent will be obtained through a signed consent form before enrollment in the study. Participants can withdraw from the study at any time during the study process, and they are informed that this has no consequence on their ongoing treatment at BFH.

The primary risk in this study is that participants may gain a false sense of reassurance, believing that they are being closely monitored. This is mitigated by informing the participants that they are not in any way monitored by health care staff during the study. This information is given at the onboarding visit and throughout the study. Participants are informed to always contact their physician if they have questions or concerns about their health. Participants may be exposed to discomfort when they wear the ECG monitor for a week. This discomfort has been mitigated by providing an ECG monitor that is small (37 × 37 × 8 mm) and lightweight (9.4 g) and can be attached to the participants’ chest using either a strap or a self-adhesive electrode. Participants may spend more time on their smartphones due to this study, yet use of the app is voluntary, and participants can choose how much time they spend on the app without any consequence.

There will be no remuneration to the participants for taking part in the study, but they will be reimbursed for direct expenses related to the study, such as travel costs.

Data management is subject to the privacy policy of the Technical University of Denmark. This study will not share personal data with anyone outside the study. Personal data will not be published. However, as part of this research, the data will be subject to processing and analysis, and the results of such analyses will be disseminated through scientific publications in academic journals, conferences, and public datasets. Moreover, any such results will be published only in anonymized formats with no person-identifiable data.

## Results

Study recruitment started in January 2025. This study is in the data collection phase. Data analysis is scheduled to start after all the data are collected. As of December 2025, we have recruited 9 participants. The Python scripts for data extraction and analysis are now available in an initial version. Results are expected to be published in 2027.

## Discussion

### Principal Findings

Several research studies have investigated the use of digital health technology for home monitoring of patients with HF, and promising results have been reported [9-14]. Telemonitoring of patients with HF is a class IIb indication according to the European Society of Cardiology guidelines [15]. Telemedicine seems to be a favorable way of patient management due to the possibility of detecting health deterioration in the early stages, especially in patients with chronic disease, where a regular follow-up is beneficial. In terms of chronic HF, the worsening of HF symptoms or, ultimately, HF decompensation could be prevented through regular telemedical follow-up visits, with the possibility of making early adjustments to the therapy and, through that, potentially preventing hospital admissions.

This study advances the field of cardiovascular disease management and digital health monitoring by facilitating the discovery of digital biomarkers based on longitudinal, ambulatory, contextual, and detailed monitoring of HF in the patients’ free-living environment. By integrating passive collection of physiological, contextual, and behavioral data with active collection of patient-reported EMAs and surveys, this study offers a nuanced understanding of heart behavior during daily activities and sleep. This dataset will provide an unprecedented insight into the everyday details of patients living with HF. In turn, this will allow us and other researchers to design and evaluate advanced data science machine learning models for the identification, classification, clustering, and prediction of HF-related problems.

This foundational data science research will promote further research into mHealth apps for monitoring based on mobile and wearable sensing, replacing traditional norms that require a patient to travel to their health care provider. Through future development and validation of predictive models for HF deterioration, this study contributes to the creation of early intervention tools that can alert health care providers and patients about potential exacerbations. This proactive approach could prevent severe episodes and hospitalizations, reducing the burden on the health care system.

### Limitations

Although the data acquired are novel and a valuable resource in learning more about contextual ECG rhythms in patients with HF, there are several limitations to this study.

First, the sample size of 40 participants, although sufficient for initial explorations of digital biomarkers, is relatively small and may not provide enough power to detect subtle associations or generalize the findings to the broader HF population. Second, participants are recruited from a single hospital in Copenhagen, which may limit the diversity of the study population and introduce selection bias. The homogeneity of the sample population may introduce difficulties when extrapolating the model to heterogeneous cohorts. Third, the reliance on technology in real-world settings introduces several challenges, including communication errors, which may lead to incomplete data capture. As participants are responsible for fitting their own ECG sensors, there is a potential for incorrect placement, which could introduce noise and artifacts in the ECG signals. Fourth, noncompliance or inconsistent use of the wearable devices or smartphone app could lead to data loss or incomplete datasets, affecting the study outcomes. Fifth, although the study design attempts to account for known confounders, there may be unmeasured variables that could influence the outcomes, such as socioeconomic factors, dietary habits, or other medical conditions. Sixth, the study relies on participant-reported measures on the app, which are subject to bias. Participants’ reports might be influenced by their current health status, their mood, or misunderstandings of the questions.

### Comparison With Prior Work

While there are other open-source datasets such as the Sleep Heart Health Study [29], they are not focused on cardiology-related outcomes and contain only polysomnographic data. The Copenhagen Center for Health Technology Contextualized Arrhythmia Database [30] is a good source of ambulatory ECG recordings, but it only has atrial fibrillation as a clinical outcome. The Telehealth Network of Minas Gerais [31] dataset contains all-cause mortality outcomes, including HF, and is used in deep learning–based data science analysis [32]. However, it only records 10-second ECG data at baseline in the clinic and not in an ambulatory manner. The Medical Information Mart for Intensive Care [33] dataset contains detailed physiological signals recorded from intensive care unit patients and includes HF events and patients but is not recorded in an ambulatory manner. The Sudden Cardiac Death in Chronic Heart Failure [34] is a dataset that contains ambulatory ECG recordings over an entire day but does not contain actimetry data or other valuable contextual information. This dataset also focuses on sudden cardiac death in end-stage HF (New York Heart Association class 3-4). There are multiple ambulatory ECG databases, such as the Beth Israel Deaconess Medical Center Congestive Heart Failure Database [35], China Physiological Signal Challenge 2021 database [36], and the Icentia11K dataset [37], but none of them contain follow-up outcomes that are needed for evaluation of HF risk. The proposed dataset from the CATCH-ECG study is, to the best of our knowledge, not available in the open-source clinical data repositories.

### Conclusions

The CATCH-ECG study aims to collect data that help design a set of digital biomarkers for recognition, classification, and prediction of HF decompensation in an ambulatory setting. The data are collected and managed by the CARP infrastructure, including the CARP Studies app used by the participants. Data collection runs for a year (52 weeks) and includes five 1-week sampling periods (quarterly assessment periods). The data collected include ambulatory monitoring of physiological (eg, ECG), contextual (eg, geolocation), and behavioral (eg, physical activity) data combined with active collection of patient-reported EMAs (eg, on sleep) and surveys (on fatigue and QoL). Data collection also includes clinical data from the participants’ medical records as well as qualitative usability data on the use of the sensor technology. The aim is to identify and publish the digital biomarkers along with an anonymized and publicly available dataset.

The potential scientific benefits of this study are substantial. By continuously monitoring heart activity using noninvasive ECG sensors and a mobile app, this study has the potential to provide a basis for developing predictive algorithms for the early detection of HF deterioration. Early detection can lead to timely interventions, reducing the need for hospital readmissions and improving patient outcomes. This proactive approach has the potential to benefit future patients by providing a model for effective HF management. Moreover, the collected data will contribute to the broader understanding of HF, aiding in the development of better treatment protocols and digital health solutions. This study is expected to significantly advance HF management by enabling early intervention and reducing health care system burden.

## Abbreviations

BFH: Bispebjerg-Frederiksberg Hospital
CARP: Copenhagen Research Platform
CATCH: Cardio-Share Model for Cross-Sectoral Ambulatory Treatment of Congestive Heart Disease Based on Personal Health Technology
CUMACF: Copenhagen Center for Health Technology Unified Methodology for Assessment of Clinical Feasibility
ECG: electrocardiogram
EMA: ecological momentary assessment
FAS: Fatigue Assessment Scale
HF: heart failure
HR: heart rate
mHealth: mobile health
QoL: quality of life
REDCap: Research Electronic Data Capture
